# Gastrointestinal disease is an important influencing factor of osteoporosis fracture:a retrospective study in chinese postmenopausal women

**DOI:** 10.1186/s12891-023-06765-4

**Published:** 2023-08-18

**Authors:** PengChao Xu, JiRong Ge, Hong Jiang, YuJuan Lin, YunJin Ye, XiaoBin Huang, YanYan He, LiPeng Xue

**Affiliations:** 1 Fujian University of Traditional Chinese Medicine, 1 Qiuyang Road, Minhou Upper Street, Fuzhou, Fujian China; 2 Fujian Key Laboratory of Integrated Traditional Chinese and Western Medicine for the Prevention and Treatment of Osteoporosis(Fujian Academy of Chinese Medical Sciences, The Affiliated Rehabilitation Hospital of Fujian University of Traditional Chinese Medicine), Fujian Academy of Chinese Medical Sciences, 282 Wusi Road, Gulou District, Fuzhou, Fujian China

**Keywords:** Osteoporosis, Osteoporotic fracture, Gastrointestinal diseases, Case control study, Chinese postmenopausal women

## Abstract

**Backgroud:**

The influencing factors of osteoporosis are complex, the incidence of osteoporosis is higher in middle-aged and elderly women, and osteoporotic fractures (OF) can seriously affect quality of life. Currently, the correlation analysis between gastrointestinal diseases and OF focuses more on diseases such as gastric cancer and inflammatory bowel disease (IBD). This study analyzed the risk factors for osteoporosis and osteoporotic fractures in 1567 postmenopausal women in Fuzhou, China. The purpose is to explore the potential influence of gastrointestinal diseases on the occurrence of OF.

**Methods:**

According to inclusion and exclusion criteria, a total of 1567 subjects were included in the analysis of OP risk factors, including 647 in the OP group and 920 in the NOP group. A total of 616 subjects were included in the analysis of correlation between OF and gastrointestinal diseases, including 132 in OF group and 484 in NF group. Statistical analysis shows that age (OR = 1.062, 95% CI = 1.045–1.080), height (OR = 0.089, 95% CI = 0.009–0.857), weight (OR = 0.981,95% CI = 0.967–0.995) and nature of work (P = 0.010) are the main risk factors for osteoporosis in postmenopausal women in southeast China, and gastrointestinal diseases (OR = 1.583, 95% CI = 1.070–2.343) and height (OR = 0.003, 95% CI  = 0.000-0.104) are the main risk factors of OF.

**Conclusions:**

The main factors affecting the occurrence of OP in postmenopausal women in southeast China are individual characteristic. Gastrointestinal diseases that do not directly affect BMD increase the risk of OF in osteoporotic patients.

## Introduction

Osteoporosis (OP) is defined as a systemic skeletal disease characterised by low bone mass and microarchitectural deterioration of bone tissue, with a consequent increase in bone fragility and susceptibility to fracture [1]. Due to the physiological characteristics of the human body, the prevalence of OP in postmenopausal women is higher than that in men of the same age [2]. As a serious consequence of OP, osteoporotic fracture is characterized by high mortality and disability rate, which significantly reduces the quality of life of patients and brings heavy economic burden [3]. Therefore, it is necessary to explore the risk factors of osteoporosis and osteoporotic fracture in postmenopausal women. In addition to the secondary osteoporosis that has been clearly and directly lead to bone loss, such as glucocorticoid treatment history, various cancers (in particular, breast cancer and cervical cancer, which mainly occur in women), thyroid diseases, inflammatory bowel disease (IBD) [4, 5]. According to the global consensus on OP and osteoporotic fracture (OF) health management, physical factors such as age, genetics, thinness can affect the onset of OP [6]. Experts agree that living situations, such as smoking, education and cognition of OP,and disease situations can also affect the occurrence of OP [7]. In addition, the concentrations of blood calcium, magnesium, and other substances are closely related to the occurrence of osteoporosis fractures [8]. Moreover, a study has found that there is a certain relationship between 25-hydroxyvitamin D levels and residential latitude [9], which indicates that region also has an important influence on the occurrence of OP.

OF are the common and most serious complications of osteoporosis. Poor bone quality, weak repair capacity, instability, and high failure rate of internal fixation are main characteristics of OF. Proximal humerus, distal radius, hip and spine are common osteoporotic fractures. The prevalence of vertebral fractures in China is about 15% in women over 50 years old [10]. It is predicted that the worldwide incidence of hip fractures will grow to 6.3 million yearly by 2050 [11]. Because the pathogenesis of OP and OF is complex and related to many factors such as individual characteristic, living situations, and disease situations, there are some differences in the incidence and influencing factors of OP and OF among people of different races and regions [12]. Although there are a large number OF risk factor analysis studies on osteoporosis and fragility fractures in the world, there are still few clinical studies on OP, OF incidence and risk factor analysis in postmenopausal women of Han nationality in southeast China. In this case-control study, individual characteristic, living situations, and disease situations were compared and analyzed in nearly 1600 postmenopausal women in Fuzhou, China. To explore the incidence and risk factors OP and OF postmenopausal women in southeast China, in order to provide evidence support for intervention and health management of OP and OF postmenopausal women in China.

Gastrointestinal diseases are highly prevalent in the Chinese population due to factors such as Chinese dietary habits:Nearly half of all new gastric cancer cases and deaths from H. pylori infection worldwide occur in China [13]. The incidence rate of chronic gastritis in China is about 20%,and the most common is chronic non-atrophic gastritis, which is nearly 50% [14]. Another study shows that [15], with the increase of age, the incidence rate of gastritis is also higher, at the same time, the incidence of osteoporosis also has the same trend, Chinese elderly women are often troubled by gastrointestinal diseases and osteoporosis. Previous studies on the correlation between gastrointestinal diseases and osteoporosis mainly focused on the diseases that have obvious effects on BMD, such as gastric cancer, post-gastrectomy, IBD [16–18]. Patients with gastric cancer who underwent gastrectomy had a significantly increased risk of OP within 10 years [19]. The incidence of OP in the elderly after gastrectomy was also significantly higher, reaching 38.2% [20]. Patients with IBD are thought to have an increased risk of bone mineral loss, resulting in osteoporosis and an increased risk of fragility fractures [21, 22]. Secondary OP and fragility fractures caused by gastrointestinal diseases have been studied extensively. Some scholars have shown through literature studies [23] that postgastrectomy, and IBD can increase the risk of bone loss and fracture. Although the correlation between these gastrointestinal diseases and OP has been recognized, it has not received much attention from the patients concerned [24], calling for the academic community to pay more attention to the health management of OP in patients with these diseases. Therefore, chronic gastrointestinal diseases that do not directly cause bone loss, such as chronic gastritis and functional gastrointestinal diseases, have less attention. Chronic gastrointestinal diseases may cause malnutrition, changes in intestinal flora, and disorders in the metabolism of trace elements, which may have a certain impact on the occurrence of OP. In addition, many scholars have noticed that proton pump inhibitor (PPI), a first-line drug for chronic gastritis, has a certain impact on the occurrence of fractures. Some scholars believe that, the possible mechanisms of PPIs leading to fracture include hyperparathyroidism caused by excessive histamine secretion and high gastrin, as well as malabsorption of minerals and vitamin B caused by hypochloremia, which may also have direct effects on bone cells [25]. In Taiwan [26] and South Korea [27, 28], there have also been clinical reports that PPI use can increase the risk of fragility fracture. Therefore, the study on the risk factors of gastrointestinal diseases and OP and OF in elderly women is also of great significance to the health management of OP, OF in elderly women and to improve the quality of life of osteoporotic patients. Based on this, this study included 1567 postmenopausal women in Fuzhou, China, to conduct a retrospective study on the risk factors of OP and OF, and focused on the impact of gastrointestinal diseases on OF.

## Materials and methods

### Study design and study population

The data of this retrospective study was collected from January 2012 to December 2021. Through telephone and leaflets, local women over 40 years old in Fuzhou were invited to carry their ID cards and medical records to the Osteoporosis Laboratory of Fujian Academy of Chinese Medical Sciences for BMD examination and questionnaire survey. A total of 2,729 people completed the questionnaire and BMD examination, and 1,567 were included in the study. All subjects were able to provide complete medical records and independently sign informed consent.

**Fig. 1:**
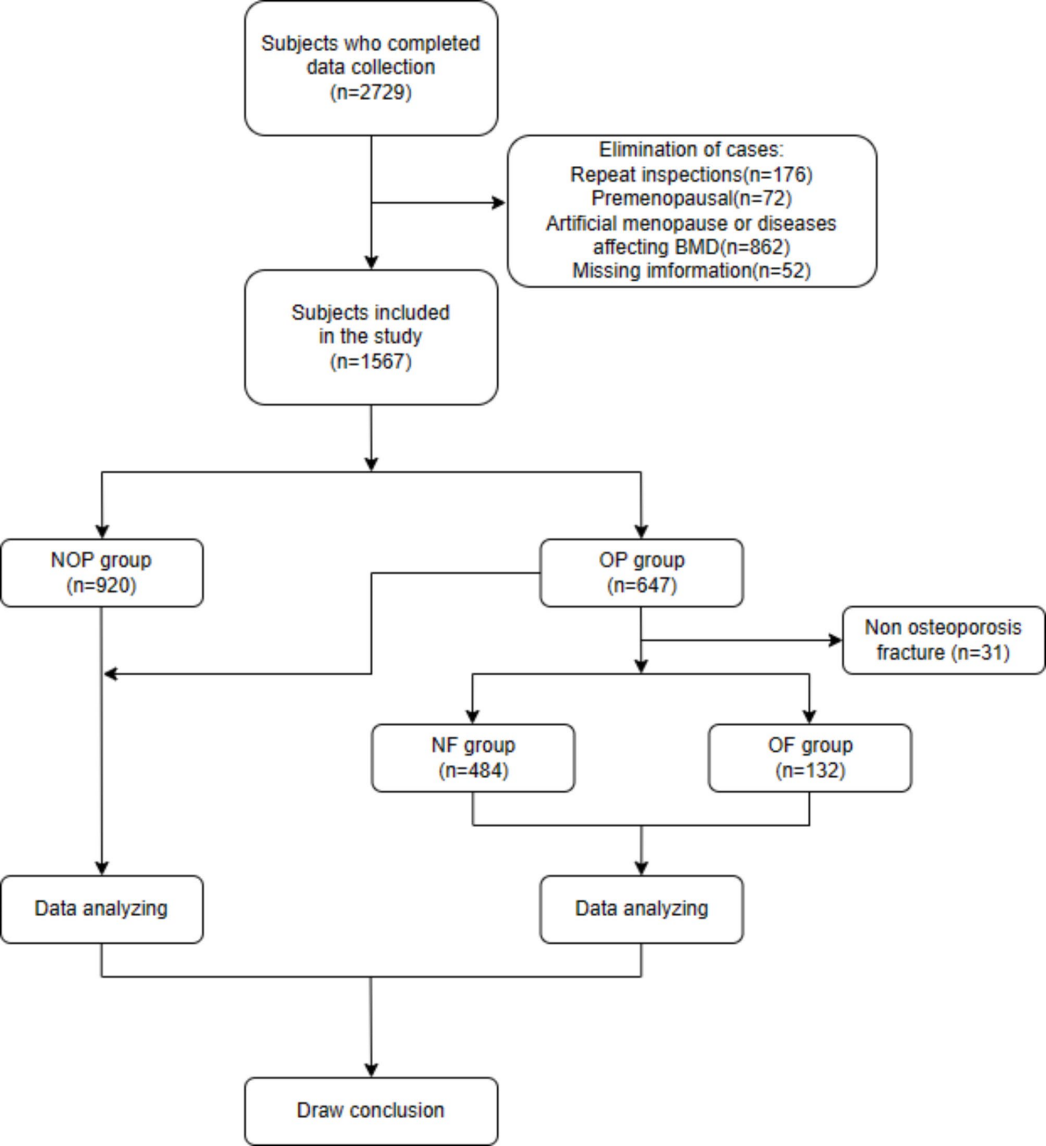
Flow diagram of this study

The inclusion criteria for this study included women aged 40–90 years old who volunteered to participate and signed informed consent from Han Chinese residents in Fuzhou. Exclusion criteria include: 1) Those who repeatedly participated in questionnaire survey and BMD examination. 2) Premenopausal or artificially menopausal women. 3) Patients with IBD, thyroid disease, rheumatoid arthritis, various kinds of cancer, serious liver, kidney or blood disease and other diseases that may cause secondary osteoporosis, and those who are receiving treatment for osteoporosis or taking other drugs that affect bone metabolism. In addition, 52 people with missing key data were excluded during data cleaning. According to the definition of osteoporosis, all subjects included in the study were divided into osteoporosis group (OP group) and non-osteoporosis group (NOP group) for osteoporosis risk factor analysis. In the OP group, 31 osteoporotic patients with non-fragility fractures were excluded according to the definition OF, and then divided into the osteoporotic fractures group (OF group) and no fracture group (NF group) for data analysis. Further details of this study are shown in flow diagram (Fig. 1).

### Clinical characteristics

The data needed for the study came from questionnaires. The questionnaire mainly includes individual characteristic, living situations, and disease situations, individual characteristic includes: age, height, weight, menopausal age, BMD and T-score of lumbar vertebrae and femoral neck, occupation, nature of work and education. Living situations includes: tea consumption, coffee consumption and milk consumption, sunlight exposure, and regular exercise. Disease situations includes: hypertension, coronary heart disease (CHD), gastrointestinal diseases and knee osteoarthritis (KOA). The subjects’ medical history were recorded by inquired about the subjects’ medical history and checked their medical records, including OF, hypertension, CHD, gastrointestinal disease and KOA. The above contents were collected by trained doctors and carefully filled in a unified questionnaire. Height and weight of each subject were measured with calibrated instruments accurate to 0.1 cm and 0.1 kg, respectively. After measuring the height and weight, calculate the body mass index (BMI) according to the formula: BMI = weight (kg) / height^2^ (m^2^).

### BMD examination

All subjects undergo unified BMD examination at our research institution. Dual-energy X-ray absorptiometry (DXA) (Discovery W, Hologic Inc., USA) was used to measure BMD of the first to fourth lumbar vertebrae and left femoral neck. The T-score was alculated according to the reference range of the instrument manufacturer. The examination is conducted by two professional doctors who are uniformly trained to reduce deviations. The coefficient of variation (CV) for repeated measurements was approximately 1.0%.

### Definitions

The diagnosis of osteoporosis was based on the T-score in the lumbar vertebrae or femoral neck. A T-score ≤ − 2.5 indicates osteoporosis, osteopenia is diagnosed as − 2.5 < T-score < − 1.0, and a T score ≥ − 1.0 indicates normal [29]. All subjects were divided into NOP group (NOP) and osteoporosis group (OP) according to T-score. Osteoporotic fractures are defined in reference to the European OP Guidelines [30]: fragility fractures of the spine, hip, distal forearm and proximal humerus following osteoporosis were recorded as osteoporotic fractures. The OP group was divided into NF group and OF group according to the occurrence of osteoporotic fracture. According to the International Statistical Classification of Diseases and Related Health Problems (ICD) (tenth revision, ICD-10). Gastrointestinal diseases included in this study: Esophagitis (ICD-10 code K20), gastritis and duodenitis (ICD-10 code K29), gastroesophageal reflux (ICD-10 code K21), gastric ulcer (ICD-10 code K25), duodenal ulcer (ICD-10 code K26), gastrojejunal ulcer (ICD-10 code K28) and peptic ulcer with unspecified site (ICD-10 code K27). The above definitions of gastrointestinal diseases refer to the Chinese medical textbook: Internal Medicine-Gastroenterology & Hepatology [31]. Other chronic diseases include: hypertension (ICD-10 code I10), CHD (ICD-10 code I25), KOA (ICD-10 code M17). Hypertension was defined as systolic blood pressure ≥ 140 mmHg and/or diastolic blood pressure ≥ 90 mmHg or intake of antihypertensive drugs. The diagnosis of coronary heart disease was based on the guidelines of the European Society of Cardiology and Chinese Society of Cardiology of Chinese Medical Association: the subjects had discomfort related to myocardial ischaemia, including location, character, duration and relationship to exertion and other exacerbating or relieving factors. All subjects had a resting 12-lead ECG recorded allowing for detection of ST-segment changes [32, 33]. The diagnosis of KOA is mainly determined by history, physical examination, and imaging studies [34], that includes knee pain, friction when moving, morning stiffness, and osteophyte at the edge of knee joint shown by X-ray. Participants were asked during the questionnaire survey and their medical records were checked to determine if they had any of the above diseases and records were kept. BMI category were generally classified into underweight (< 18.5 kg/m^2^), normal weight (18.5 ~ 23.9 kg/m^2^), overweight (24 ~ 27.9 kg/m^2^), and obese (≥ 28 kg/m^2^) in accordance with the criteria of the Working Group on Obesity in China [35]. In terms of daily drinking, 200ml is defined as the limit and divided into three groups: no drinking, occasional drinking (0-200ml), regular drinking (> 200ml/day). According to China’s national conditions, the occupations of participants are divided into five categories: farmers, workers, cadres, intellectuals and others; The education level is divided into below junior high school group, below bachelor degree group and bachelor degree or above.

### Statistical analysis

EpiDate 2 (the EpiData Association, Odense M, Denmark) was used to enter and proofread data repeatedly, and SPSS 26 (IBM, Inc., New York, USA) software was used for data analysis. The results are expressed as the average standard deviation or quantity (percentage). Independent sample t test or Mann-Whitney U test were used according to whether continuous variables conform to normal distribution. Pearson chi-square test and Fisher exact probability method were used to measure the difference in frequency. Logistic regression analysis was carried out with OP and OF as dependent variables to investigate the factors affecting OP and OF. First, the independent variables were screened by P > 0.2. Univariate regression analysis was used to exclude variables without statistical significance and eliminate mediary variables. Multicollinearity was assessed using stepwise regression. For continuous variables, the linear condition was assessed. The − 2 log-likelihood ratio was used to test the overall significance of the model. The HosmerLemeshow test evaluated the model’s goodness-of-fit. Except screening the independent variables, all statistical hypothesis tests were two-sided and performed at the 0.05 significance level.

## Results

A total of 1,567 subjects were enrolled after excluding 1,162 subjects who did not meet the criteria.Further details are shown in flow diagram (Fig. 1). Statistically, subjects in the OP group had a higher mean age than those in the NOP group, while height, weight, and menopause age were significantly smaller than those without osteoporosis (P < 0.001). In addition, BMD in OP group was significantly lower than that in NOP group (P < 0.001) (Table 1).

**Table 1:** Demographic data with participants.(mean ± SD)

	Total (n = 1567)	Osteoporosis (n = 647)	Non-osteoporosis (n = 920)	P value	Number of missing sample data
**Age** (years)	62.86±6.80	64.54 ± 6.83	61.69 ± 6.53	**0.000**	0
**Height** (m)	1.558 ± 0.05	1.548 ± 0.06	1.565 ± 0.05	**0.000**	0
**Weight** (kg)	56.39 ± 8.45	56.36 ± 8.21	58.12 ± 8.55	**0.000**	0
**BMI** (kg/m^2^)	23.62 ± 3.09	23.50 ± 3.06	23.71 ± 3.11	0.205	0
**Menopause age** (years)	50.35 ± 3.60	49.92 ± 3.69	50.65 ± 3.51	**0.000**	0
**Lumbar vertebra BMD** (g/cm2)	0.781 ± 0.15	0.660 ± 0.08	0.867 ± 0.13	**0.000**	4
**Femoral neck BMD** (g/cm2)	0.696 ± 0.13	0.631 ± 0.12	0.743 ± 0.11	**0.000**	11

statistical method: Mann-Whitney U-test

The OF group and NF group were grouped based on 647 subjects in the OP group. After excluding 31 subjects with non-osteoporotic fractures, 616 subjects were included in this study. The data in Table 2 show the baseline of OF group and NF group. The age of NF group was lower (P < 0.001) and higher height (P < 0.002), the femoral neck had higher BMD (P < 0.001).

**Table 2:** Demographic data with osteoporotic fracture group and no fracture group.(mean ± SD)

	Total (n = 616)	Osteoporotic fracture (n = 132)	Osteoporosis without fracture (n = 484)	P value	Number of missing sample data
**Age** (years)	64.72 ± 6.81	68.17 ± 7.19	63.79 ± 6.40	**0.000** ^a^	0
**Height** (m)	1.548 ± 0.06	1.534 ± 0.06	1.551 ± 0.05	**0.002** ^b^	0
**Weight** (kg)	56.41 ± 8.15	55.16 ± 8.12	56.75 ± 8.13	0.079^a^	0
**BMI** (kg/m^2^)	23.53 ± 3.03	23.41 ± 3.05	23.56 ± 3.03	0.615^a^	0
**Menopause age** (years)	49.86 ± 3.71	49.47 ± 3.92	49.96 ± 3.66	0.216^a^	0
**Lumbar vertebra BMD** (g/cm2)	0.660 ± 0.08	0.652 ± 0.09	0.662 ± 0.08	0.689^a^	2
**Femoral neck BMD** (g/cm2)	0.631 ± 0.12	0.584 ± 0.11	0.644 ± 0.12	**0.000** ^a^	4

a: Mann-Whitney U-test, b: independent t-test

Among the 132 subjects, 37 (28.03%) suffered spinal fracture, 13 patients with humerus fracture (9.85%). there were 40 patients with ulna and radius fracture (30.30%), 8 patients with hip fracture (6.06%), 25 patients with other lower limb fractures (including tibia, calcaneus and so on) (18.94%), and 9 patients with multiple site fractures (6.82%).

### Comparison of influencing factors between OP group and NOP group

Individual characteristic, living situations, and disease situations of the two groups were preliminarily compared, and the preliminary screening of risk factors was conducted according to the results, as shown in Table 3. Set p < 0.2 is the factor that may affect OP. The risk factors included in the analysis were age, height, weight, nature of job, education, tea consumption, coffee consumption, milk consumption and CHD. The above factors were analyzed by univariate and multivariate regression analysis, and the adjusted results showed that the increase of age was a risk factor for OP, the increase of height and weight could reduce the occurrence of OP, and the nature of work may also affect the occurrence of OP (Table 4).

**Table 3:** Comparison of individual characteristic, living situations and disease situations influencing factors between the osteoporosis group and non-osteoporosis group

Variable	Osteoporo-sis (n,%)	Non-osteo-porosis(n,%)	Χ^2^ test	P value	Number of missing sample data
**Individual characteristic**					
**BMI**					
Underweight	25,45.5%	30,54.5%	2.758	0.430	0
Normal	366,42.6%	494,57.4%			
Overweight	202,38.5%	323,61.5%			
Obese	54,42.5%	73,57.5%			
**Occupation**					
Farmer	40,50.6%	39,49.4%	3.215	0.522	3
Worker	188,40.0%	282,60.0%			
Cadre	99,41.2%	141,58.8%			
Intellectual	136,41.6%	191,58.4%			
Others	183,40.8%	265,59.2%			
**Nature of work**					
Manual labour	212,42.2%	290,57.8%	5.329	**0.070**	0
Mental labour	318,39.0%	498,61.0%			
Both above	117,47.0%	132,53.0%			
**Education**					
Below junior high school	297,44.3%	374,55.7%	5.960	**0.051**	0
Below bachelor degree	310,39.9%	467,60.1%			
Bachelor degree or above	40,33.6%	79,66.4%			
**Living Situations**					
**Tea consumption**					
No or drinking occasional	560,41.0%	805,59.0%	4.679	**0.096**	3
0−200ml/day	43,51.8%	40,48.2%			
>200ml/day	43,37.1%	73,62.9%			
**Coffee consumption**					
No or drinking occasional	630,41.3%	894,58.7%	3.784	**0.151**	1
0−200ml/day	5,25.0%	15,75.0%			
>200ml/day	12,54.5%	10,45.5%			
**Milk consumption**					
No or drinking occasional	366,39.2%	567,60.8%	4.269	**0.118**	1
0−200ml/day	61,46.6%	70,53.4%			
>200ml/day	219,43.6%	283,56.4%			
**Sunlight exposure**					
<1 h/day	424,41.2%	604,58.8%	0.559	0.756	5
1−3 h/day	209,41.8%	291,58.2%			
>3 h/day	12,35.3%	22,64.7%			
**Regular exercise**					
No	241,41.2%	344,58.8%	0.003	0.954	13
Yes	406,41.3%	576,58.7%			
**Disease Situations**					
**Hypertension**					
*No*	450,40.6%	659,59.4%	0.792	0.673	2
*No examination*	25,43.9%	32,56.1%			
*Yes*	171,42.9%	228,57.1%			
**CHD**					
*No*	570,40.5%	837,59.5%	3.911	**0.141**	0
*No examination*	29,51.8%	27,48.2%			
*Yes*	48,46.2%	56,53.8%			
**Gastrointestinal diseases**					
*No*	385,40.9%	557,59.1%	0.171	0.679	0
*Yes*	262,41.9%	363,58.1%			
**KOA**					
*No*	327,41.4%	462,58.6%	0.024	0.988	3
*No examination*	194,41.0%	279,59.0%			
*Yes*	125,41.4%	177,58.6%			
**Fracture**					
*No*	483,37.8%	795,62.2%	34.291	**0.000**	1
*Yes*	163,56.6%	125,43.4%			

statistical method: Pearson Chi-Square

**Table 4:** Multivariate Logistic regression analysis for the effect of independent variables on osteoporosis

	Univariate Logistic regression					Multivariate Logistic regression			
	B	Crude OR	95%CI	*P*		B	Adj. OR	95%CI	*P*
**Age**	−4.364	1.066	1.049–1.082	**<0.001**		0.060	1.062	1.045–1.080	**<0.001**
**Height**	−5.818	0.003	0.000−0.020	**<0.001**		−2.422	0.089	0.009–0.857	**0.037**
**Weight**	−0.025	0.975	0.963–0.987	**<0.001**		−0.019	0.981	0.967–0.995	**0.007**
**Nature of work**				0.070					**0.010**
*Manual labour*									
*Mental labour*	−0.135	0.873	0.697–1.095	0.241		−0.198	0.821	0.648–1.039	0.104
*Both above*	0.193	1.212	0.893–1.646	0.216		0.252	1.287	0.939–1.765	0.117
**Education**				0.052					
*Below Junior high school*									
*Below bachelor degree*	−0.179	0.836	0.678–1.031	0.093					
*Bachelor degree or above*	−0.450	0.638	0.423–0.960	**0.031**					
**Tea**				0.099					
*No or drinking occasional*									
*0−200ml/day*	0.435	1.545	0.991–2.409	0.055					
*>200ml/day*	−0.166	0.847	0.572–1.253	0.405					
**Coffee**				0.161					
*No or drinking occasional*									
*0−200ml/day*	-0.749	0.473	0.171–1.308	0.149					
*>200ml/day*	0.532	1.703	0.731–3.966	0.217					
**Milk**				0.119					
*No or drinking occasional*									
*0−200ml/day*	0.300	1.350	0.935–1.950	0.110					
*>200ml/day*	0.181	1.199	0.962–1.494	0.106					
**CHD**				0.144					
*No*									
*No examination*	0.456	1.577	0.924–2.693	0.095					
*Yes*	0.230	1.259	0.844–1.878	0.260					

### Comparison of influencing factors between OF group and NF group

In the analysis of these two groups, we first analyzed the association of osteoporotic fractures with chronic disease. Through analysis, we found that gastrointestinal diseases may have an impact on the OF occurrence (Table 5). We adjusted the regression coefficient between gastrointestinal diseases and OF by further comparing factors such as individual characteristic, living situations. Through screening, the main influencing factors included in the analysis are age, height, weight, occupation, tea consumption, milk consumption and regular exercise (Table 6). Before the regression analysis, age, a mediary variable that could increase the incidence of gastrointestinal diseases, OF and OP, was excluded, and the OR value of gastrointestinal diseases to OF was adjusted through the remaining influencing factors, and the regression equation only included height and gastrointestinal diseases. After adjusting, the OR value OF gastrointestinal diseases for OF was 1.583, p = 0.022, indicating that gastrointestinal diseases can significantly improve the risk of OF. That gives us a new hint of health management and education for osteoporosis patients in the future (Table 7).

**Table 5:** Comparison of disease situations influencing factors between the osteoporotic fracture group and no fracture group

Variable	Osteoporotic fracture (n,%)	Osteoporosis without fracture(n,%)	Χ^2^ test	P value	Number of missing sample data
Hypertension					
*No*	83,19.6%	341,80.4%	2.876	0.237	1
*No examination*	5,20.0%	20,80.0%			
*Yes*	43,25.9%	123,74.1%			
**CHD**					
*No*	115,21.2%	427,78.8%	0.819	0.664	0
*No examination*	5,17.9%	23,82.1%			
*Yes*	12,26.1%	34,73.9%			
**Gastrointestinal diseases**					
*No*	68,18.4%	301,81.6%	4.920	**0.027**	0
*Yes*	64,25.9%	183,74.1%			
**KOA**					
*No*	69,22.5%	237,77.5%	1.361	0.506	1
*No examination*	35,18.4%	155,81.6			
*Yes*	27,22.7%	92,77.3%			

statistical method: Pearson Chi-Square

**Table 6:** Screening of risk factors for osteoporotic fractures

Variable	Osteoporotic fracture (n,%)	Osteoporosis without fracture(n,%)	Χ^2^ test	P value	Number of missing sample data
BMI					
*Underweight*	7,31.8%	15,68.2%	2.987	0.393^b^	0
*Normal*	78,22.3%	272,77.7%			
*Overweight*	35,18.2%	157,81.8%			
*Obese*	12,23.1%	40,76.9%			
**Occupation**					
*Farmer*	13,33.3%	26,66.7%	6.449	**0.168** ^**a**^	1
*Worker*	39,21.5%	142,78.5%			
*Cadre*	18,19.1%	76,80.9%			
*Intellectual*	32,25.2%	95,74.8%			
*Others*	30,17.2%	144,82.8%			
**Nature of work**					
*Manual labour*	41,20.3%	161,79.7%	2.378	0.304^a^	0
*Mental labour*	71,23.8%	227,76.2%			
*Both above*	20,17.2%	96,82.8%			
**Education**					
*Below Junior high school*	58,20.5%	225,79.5%	0.456	0.796^a^	0
*Below bachelor degree*	67,22.6%	230,77.4%			
*Bachelor degree or above*	7,19.4%	29,80.6%			
**Tea consumption**					
*No or drinking occasional*	122,22.8%	412,77.2%	4.679	**0.085** ^a^	1
*0−200ml/day*	6,15.0%	34,85.0%			
*>200ml/day*	4,9.8%	37,90.2%			
**Coffee consumption**					
*No or drinking occasional*	130,21.7%	469,78.3%	0.846	0.786^b^	0
*0−200ml/day*	0,0.0%	5,100.0%			
*>200ml/day*	2,16.7%	10,83.3%			
**Milk consumption**					
*No or drinking occasional*	66,18.9%	284,81.1%	4.269	**0.118** ^**a**^	1
*0−200ml/day*	18,29.5%	43,70.5%			
*>200ml/day*	48,23.5%	156,76.5%			
**Sunlight exposure**					
*<1 h/day*	88,22.0%	312,78.0%	0.622	0.758^b^	2
*1−3 h/day*	42,20.6%	162,79.4%			
*>3 h/day*	1,10.0%	9,90.0%			
**Regular exercise**					
*No*	56,24.6%	172,75.4%	2.110	**0.146** ^**a**^	0
*Yes*	76,19.6%	312,80.4%			

a: Pearson Chi-Square, b: Fisher Exact Test

**Table 7:** Multivariate Logistic regression analysis for the effect of independent variables on osteoporotic fractures

	Univariate Logistic regression					Multivariate Logistic regression			
	B	Crude OR	95%CI	*P*		B	Adj. OR	95%CI	*P*
**Gastrointestinal diseases**	0.437	1.548	1.051–2.281	**0.027**		0.460	1.583	1.070–2.343	**0.022**
**Height**	−5.648	0.004	0.000−0.121	**0.002**		−5.838	0.003	0.000−0.104	**0.001**
**Weight**	−0.025	0.976	0.952−1.000	**0.047**					
**Occupation**				0.177					
*Farmer*									
*Worker*	−0.599	0.549	0.258–1.168	0.119					
*Cadre*	−0.747	0.474	0.204–1.098	0.082					
*Intellectual*	−0.395	0.674	0.310–1.465	0.319					
*Others*	−0.875	0.417	0.192–0.903	**0.026**					
**Tea**				0.098					
*No or drinking occasional*									
*0−200ml/day*	−0.518	0.596	0.244–1.453	0.255					
*>200ml/day*	−1.008	0.365	0.128–1.045	0.060					
**Milk**				0.121					
*No or drinking occasional*									
*0−200ml/day*	0.588	1.801	0.977–3.322	0.059					
*>200ml/day*	0.281	1.324	0.870–2.015	0.190					
**Regular exercise**	−0.290	0.748	0.505–1.107	0.147					

## Discussion

With the continuous progress of aging population in China, the occurrence of OP and OF in our country has become an important national health problem. This retrospective study showed that the prevalence of osteoporosis and osteoporotic fracture in postmenopausal women in Fuzhou was 41.29% and 20.40% respectively. Accroding to the incidence in Fuzhou, the occurrence of OP and OF does have an important impact on the life of local elderly women. In comparison of baseline data between the groups, it was interesting to note that femoral neck BMD in the OF group was significantly lower than that in the NF group, while lumbar vertebrae BMD was not significantly different (Table 2). From the clinical perspective, a large number of elderly women find themselves suffering from osteoporosis, the initial symptoms were mostly waist pain. In terms of bone structure, the lumbar vertebrae had a higher proportion of cancellous bone, but the data here showed a more pronounced change in femoral neck BMD. The cause of this problem may be related to waist strain and osteophyma. Besides, hip fracture can greatly affect the athletic ability and mental state of the elderly [36, 37]. In previous concepts, it was often described as the dying fracture of the elderly. Combined with the results of this study, femoral neck BMD is a greater accuracy in the prevention of OF, and regular femoral neck BMD examination is more necessary in osteoporotic patients.

In the first part, the analysis results of OP show that the risk factors of OP in postmenopausal women in southeast China mainly include age, height, weight and nature of work. In OP health management and education for elderly women, we should remind older women, shorter height and lighter weight to pay more attention to the prevention and treatment of OP. In terms of the influence of living situations on OP, the impact of occupation and educational level on osteoporosis may be related to the economic status of the subjects. In addition, there may be some relationship between the amount of tea consumed, sunlight exposure, cardiovascular disease and BMD, but the statistical difference in this study is not significant. In the studies of other scholars, the effect of tea consumption on bone mineral density is still inconclusive [38–40], but sunlight exposure [41] and cardiovascular disease [42–44] may both have an impact on the prevalence of osteoporosis. In general, personal physical quality still has a greater impact on the occurrence of OP. Therefore, in the prevention and treatment education of OP, We should focus on this part of the population.

According to the analysis results of OF in the second part. We suggest that we should pay more attention to the impact of gastrointestinal diseases on OF. With the progress of an aging society, an increasing number of middle-aged and elderly people are also suffering from gastrointestinal diseases and osteoporosis. The stomach and intestines are the main organs for ingesting nutrients and maintaining human life activities. The normal function is closely related to the health status of the human body. With the growth of age, the function of the digestive system gradually weakens, and the prevalence rate of gastrointestinal diseases is also increasing [45]. This often leads to weight loss, weakness, and other issues in the elderly. In addition, with the deterioration of physical functions, muscles begin to lose, and the motor function of the elderly is gradually declining [46]. The frequent occurrence of gastrointestinal diseases and the decline in the health status of the elderly lead to a decrease in their musculoskeletal mass. These problems directly affect the BMD and bone quality of the elderly, and even lead to fragility fractures.

Because of Chinese dietary habits and not used to separate meals, the prevalence of Helicobacter pylori infection and gastrointestinal diseases are very high in China [13]. Nutritional issues or calcium phosphorus metabolism abnormalities caused by gastrointestinal diseases often lead to secondary osteoporosis and the occurrence of OF [47]. Numerous studies have confirmed the importance of these factors in the occurrence of OF. For example, the effects of gastrectomy on the nutritional status of the elderly [20], the effects of anti-breast cancer drugs on bone metabolism [5], and the effects of IBD on bone mineral loss [23] were mentioned above. However, doctors have not paid enough attention to gastrointestinal diseases that do not directly cause bone loss. The patients included in this study were excluded from various gastrointestinal diseases that directly caused bone loss, including various cancers, post-gastrectomy, and IBD. Through statistical analysis, however, our study suggests that even diseases that have little direct impact on BMD may significantly increase the risk of fracture in osteoporotic patients. At present, there are not many studies on the relevant mechanism of this condition, but in the process of sorting out relevant literatures, we still found some relevant studies [24]: A review summarizes possible pathways by which PPI affects bone metabolism, PPI may affect BMD through histamine secretion and hyperparathyroidism. But in addition to its effect on BMD, could gastrointestinal diseases contribute to the predisposition of osteoporotic patients by affecting bone mass or nerve or muscle function? Whether the physical and mental state of the elderly with gastrointestinal diseases more lead to the occurrence of OF, and what is its biological mechanism? This question deserves further study.

The results of this study suggest that when OP occurs, we should take drug intervention as early as possible, and change the diet structure and lifestyle to have limited effect on the occurrence of OP. To avoid the occurrence of OF as much as possible, we should focus on patients with chronic gastrointestinal disease and check BMD of the femoral neck regularly. Provide more targeted health education and attention to patients with gastrointestinal diseases. Of course, this study also has some shortcomings: 1) Since no accurate incidence of osteoporotic fractures has been reported in the literature in China, this study uses the data of spinal fragile fractures in the estimation of sample size. As a result, there may be some error between the calculated sample size and the actual required sample size. Furthermore, in order to increase the sample size, this study has a long time span. 2) As a single-center retrospective study, it is difficult to exclude some confounding factors, especially the influence of different regions on OP and OF, and it is also difficult to obtain a direct causality between gastrointestinal diseases and OF. 3) In the data collection of large samples, although we try our best to obtain objective data, there may be some selection bias and recall bias. In future studies, we may consider using multi-center, large sample, short-term or prospective cohort studies to further explore the relationship between gastrointestinal diseases and OF, or further explore the possible mechanism of chronic gastrointestinal diseases affecting the occurrence OF, with a view to improving the quality of life and health management of OP and OF patients.

## Conclusions

In general, the main factors affecting the occurrence of OP in postmenopausal women in southeast China are individual characteristic. Living situations and disease situations may have some influence on the occurrence of OP, but their effects are limited. Our study shows that there is a close relationship between gastrointestinal diseases and the occurrence OF, and even gastrointestinal diseases that do not directly affect bone density can increase the incidence of OF. Therefore, health education of OP and OF should be emphasized in patients with gastrointestinal diseases to reduce the occurrence of adverse events.

## Data Availability

The datasets generated and/or analysed during the current study are not publicly available due to personal protection laws. Subsets or aggregation of these data will not include information that could compromise research participants’ privacy and are available from the corresponding author on reasonable request.
